# Note from the Editor

**Published:** 2009-05

The past year has been an important time for *EHP* as we have focused on rebuilding the journal and its outreach programs. We appreciate the strong support you, our readers, have shown us. We, in turn, are committed to making *EHP* as strong as it can be, with new program features to carry our content further than ever.

This spring we are pleased to announce the launch of our new podcast series, “The Researcher’s Perspective.” Our inaugural podcast is an interview with Kristie Ebi, who discusses her recent paper “Climate Change, Tropospheric Ozone and Particulate Matter, and Health Impacts” [*Environ Health Perspect* 116:1449–1455 (2008)]. We are also putting the *EHP* Science Education Program on a brief sabbatical while we conduct a thorough evaluation to guide us in best meeting the needs of our users. We will not be publishing new lessons during this period, but instead will be busy conducting focus groups, surveys, and resource needs analyses, all with an eye toward broadening the impact of the program both domestically and internationally. Finally, in summer 2009 we will debut the all-new *EHP* website (www.ehponline.org). The site will be packed with new features, including enhanced search capabilities and a “Comment” feature that will let you discuss news articles online.

As always, we welcome your feedback and thank you for your support.

